# DNA-functionalized silicon nitride nanopores for sequence-specific recognition of DNA biosensor

**DOI:** 10.1186/s11671-015-0909-0

**Published:** 2015-05-01

**Authors:** Shengwei Tan, Lei Wang, Jingjing Yu, Chuanrong Hou, Rui Jiang, Yanping Li, Quanjun Liu

**Affiliations:** State Key Laboratory of Bioelectronics, School of Biological Science and Medical Engineering, Southeast University, No. 2 Sipailou, Nanjing, 210096 People’s Republic of China; Department of Automation, Tsinghua University, No. 1 Tsinghua Yuan, Beijing, 1000084 People’s Republic of China; Jiangxi-OAI Joint Research Institute, Nanchang University, No. 235 Nanjing Dong Lu, Nanchang, 330047 People’s Republic of China

**Keywords:** Sequence-specific recognition, DNA hybridization, Silicon nitride nanopores, Biosensor

## Abstract

Nanopores have been proven to be novel and versatile single-molecule sensors for individual unlabeled biopolymer detection and characterization. In the present study, a relatively large silicon nitride (Si_3_N_4_) nanopore with a diameter of approximately 60 nm was fabricated successfully using a focused Ga ion beam (FIB). We demonstrated a simple *ex situ* silanization procedure to control the size and functionality of solid-state nanopores. The presented results show that by varying the silanization time, it is possible to adjust the efficiency of probe molecule attachment, thus shrinking the pore to the chosen size, while introducing selective sensing probes. The functionalization of nanopores was verified by analysis of field-emission scanning electron microscopy (FESEM), energy-dispersive X-ray spectroscopy (EDS), and electrical measurements. Based on this study, we envision that the functionalized silicon nitride nanopores with the DNA probe might provide a biosensing platform for the detection and discrimination of a short single-stranded DNA oligomer of unknown sequences in the future.

**Electronic supplementary material:**

The online version of this article (doi:10.1186/s11671-015-0909-0) contains supplementary material, which is available to authorized users.

## Background

Nanopores have been widely evolved in various devices, which cover many different areas from single-molecule stochastic sensing [1-4] to medical screening and diagnosis [5], so they play an increasingly important role at the forefront of biotechnology and life science. Nanopore-based devices, both biological and synthetic, allow us to detect and interrogate single molecules by monitoring the modulation of the pore electrical conductance. This simple transduction mechanism is particularly powerful due to several particular advantages: the needed volume and concentration of the sample are reduced; there is great potential for label-free single-molecule sensing applications; and the real-time effectiveness greatly decreases the time and cost of the analysis [6]. What is more, there is widespread concern on nanopores as a potential candidate to achieve the ‘$1,000 genome’ goal set by the US National Institutes of Health [7]. Based on all these advantages, such sensors have already been successfully employed to analyze nanoparticles [8-10] and to study protein folding or unfolding [3,11]. Besides, they also have the potential to detect anomalies in a DNA strand which can give vital forewarning to doctors about a patient’s susceptibility to specific diseases like cancer. To achieve this goal, inner nanopore wall functionalization is essential to selective DNA translocation [12].

At present, a chemically modified or functionalized nanopore is considered to be a great candidate for biomolecule sensing [13]. More attention is focused on the chemically modified nanopore, which is expected to have a major impact on the bioanalysis and fundamental understanding of nanoscale chemical interactions at the single-molecule level [14]. Various approaches have been developed to modify the surface charge properties of nanochannels, including the deposition of metals [15,16] and oxides [17,18] and various organic modifications [19,20]. Asghar et al. have employed a pulsed plasma polymerization process to reduce nanopore diameters, in a highly controlled fashion [21]. Mussi and colleagues have controlled the size and functionality of solid-state nanopores based on an initial vapor-phase silanization [6], and ‘DNA-Dressed Nanopore’, as a novel class of selective biosensor devices, has been chemically functionalized by adding probe oligonucleotides [22,23]. Meller and coworkers described two approaches for monolayer coating of nanopores: (1) self-assembly from solution, in which nanopores with a 10-nm diameter can be reproducibly coated, and (2) self-assembly under voltage-driven electrolyte flow, in which they were able to coat 5-nm nanopores [24]. Prabhu’s group separated 22- and 58-nm polystyrene nanoparticles in solution using a chemically modified nanopore [25]. Chemical surface modifications were investigated and optimized by Freedman in order to detect the folded or unfolded states of bovine serum albumin (BSA) [26]. Studies of Kim et al. have shown that the surface of the nanopore was derivatized with γ-aminopropyltriethoxysilane, which attracts DNA molecules by the positively charged surface [27]. These synthesized nanopores possess adjustable size, shape, and surface properties and can be integrated into complex lab-on-a-chip devices, hence enhancing their technological applicability. Nevertheless, they do not have the function of sequence-specific recognition.

In recent years, the nucleic acid hybridization technique has been proven to be a potential platform to create biosensing devices for rapid and low-cost detection of DNA sequences. A single-stranded DNA probe was immobilized onto the sensor surface, and subsequently, recognition of a target DNA strand with sequence complementary to the tethered nucleotide was achieved via hybridization. Upon DNA/DNA duplex formation, the sensor directly transduces the hybridization events into a measurable electronic read-out signal [28,29].

**Figure 1:**
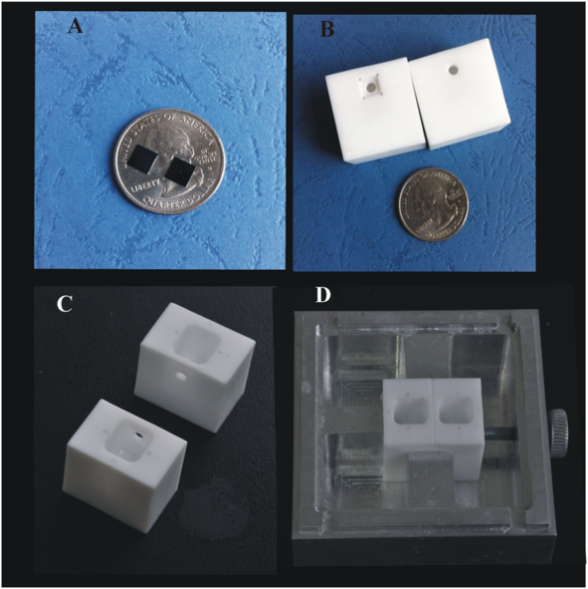
Si_3_N_4_ and modified chips. **(A)** Si_3_N_4_ chips. **(B-D)** Modified chips were carried out in the nanofluid developed in our lab to investigate their electrical properties.

To achieve this goal, a relatively large Si_3_N_4_ nanopore with a diameter of approximately 60 nm was fabricated successfully using focused ion beam (FIB). We introduced the *ex situ* approach to functionalize the nanopore as a specific recognition sensor. 3-Aminopropyltriethoxysilane (3-APTES) was selected to activate the entire membrane thanks to its high reactivity [26]. A three-step procedure based on *ex situ* silanization was adopted and illustrated in Figure 1 (details are given in the ‘Chemically modifying nanopores’ section). The functionalized nanopore has been characterized by field-emission scanning electron microscopy (FESEM), energy-dispersive X-ray spectroscopy (EDS), and current-voltage (*I*-*V*) curve measurement. The silanization time was optimized to control the size of the nanopore. The hybridization experiment was performed with target molecules both non-complementary and perfectly complementary to probe oligonucleotides. This simple preparation method can be performed in most laboratories and is applicable to many different probe molecules, showing a very powerful and versatile capability in the field of single-molecule sensing.

## Methods

### Chemicals and materials

3-APTES, potassium chloride (KCl), methanol, and 1,4-phenylene diisothiocyanate were purchased from Sigma-Aldrich (St. Louis, MO, USA). Dimethyl sulfoxide was brought from Biosharp (Hefei New Source Biological Technology Co., Ltd., Hefei, China). The DNA probe was purchased from Invitrogen Biotechnology Co., Ltd. (Carlsbad, CA, USA). The probe used for functionalization was amino-terminated at its 5′ position. The piranha solution was H_2_O_2_:H_2_SO_4_ 1:3 (*V*/*V*). All solutions were prepared with ultrapure water from a Milli-Q water purification system (resistivity of 18.2 MΩ.cm, 25°C; Millipore Corporation, Billerica, MA, USA) and filtered through a 0.02-μm Anotop filter (Whatman).

### Nanopore fabrication

**Figure 2:**
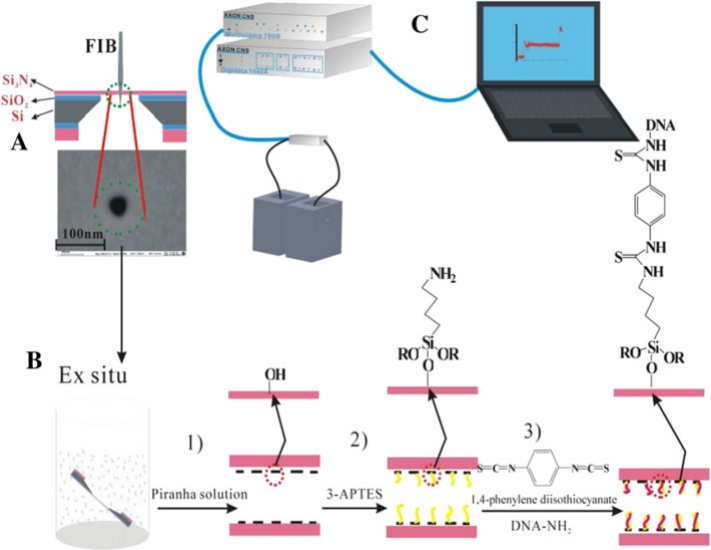
Schematic representation. **(A)** Nanopore fabrication, **(B)** functionalization of a nanopore by *ex situ* method, and **(C)** electrical measurement setup.

A free-standing membrane was obtained by depositing a thin (100-nm nominal thickness) Si_3_N_4_ film on a 300-μm-thick Si substrate. Fabrication of this membrane consisted of first depositing a layer of low-stress silicon nitride on a silicon wafer using low-pressure chemical vapor deposition (the deposition rate was 5 nm/min, chamber pressure was about −4 mbar, and the substrate temperature was 810°C) followed by photolithography (the opening window size for photolithography is 500 μm × 500 μm), deep reactive ion etching, and tetramethylammonium hydroxide (TMAH) etching to form a 50 × 50 μm^2^ square membrane. (TMAH was used for silicon etching, and the Si_3_N_4_ was used as etch mask against TMAH etching. The etch rate was about 40 μm/h, and the Si/Si_3_N_4_ etch selectivity was greater than 1,000. DRIE was used to etch Si_3_N_4_ (500 μm × 500 μm), then the 5% TMAH etching was used for silicon etching at 80°C. The silicon wafer was of the <100> type.) The nanopore was drilled on the surface of the membrane by bombarding the surface with Ga^+^ ions using a FEI Strata 201 FIB system (FEI Co., Hillsboro, OR, USA) at an acceleration potential of 30 kV while the current was measured as 1 pA. The milling time was 1.5 s under spot mode. The chip is shown in Figure 1A. The resulting nanopore was imaged by FESEM (Figure 2A).

### Chemically modifying nanopores

A schematic of the experimental procedure is given in Figure 2A. Prior to chemical modification, the chips were cleaned in piranha solution at 90°C for 30 min to produce hydroxyl groups on the Si_3_N_4_ surface. The hydroxyl groups (-OH), generated on the channel surface during the chemical reaction process, were modified with the amine-terminated DNA probe by the following procedures: firstly, the entire silicon nitride surface was covered with -OH groups on the Si_3_N_4_ surface, which was activated with 3-APTES (1% *V*/*V* in methanol) for 3 h at room temperature. Subsequently, the chip was treated with 1,4-phenylene diisothiocyanate (0.5% *W*/*V* in dimethyl sulfoxide) cross-linker for 5 h followed by two washes in dimethyl sulfoxide and two washes in double-distilled water. Finally, an overnight treatment at 37°C with 100 nM amino-modified DNA (5′-TTTTTTTTTTTTGTCATTATGTGCTGCCATATCTACTTCAGA-3′ sequence) in ddH_2_O pH 8 was performed, followed by two washes (30 min each) with 28% ammonia solution and two washes with ddH_2_O (15 min each) to deactivate the substrate. At the same time, the control experiments were carried out to demonstrate that a decrease in current actually stems from the specific surface chemistry. Firstly, the entire silicon nitride surface was covered with -OH groups on the Si_3_N_4_ surface, which was placed in methanol (1 mL) for 3 h at room temperature. Subsequently, the chip was treated with dimethyl sulfoxide (1 mL) for 5 h followed by two washes in dimethyl sulfoxide and two washes in ddH_2_O. Finally, an overnight treatment at 37°C with ddH_2_O (1 mL) pH 8 was performed, followed by two washes (30 min each) with 28% ammonia solution and two washes with ddH_2_O (15 min each) to deactivate the substrate.

### Electrical measurements

A schematic of the experimental setup is presented in Figure 2C. To perform electrical measurements, both before and after the functionalization procedure, the nanopore chips were held in place using a custom-built polycarbonate flow cell with PDMS gaskets to assure that the only path of the ionic current was through the nanopore (Figure 1B,C,D). The cell was made of two facing Plexiglas chambers filled with filtered 1 M KCl ionic solution. (Because of the high surface-to-volume ratio in nanopores, surfaces potentially have a large effect on the conductance at lower salt concentrations [30]. Besides, the four nucleobases were better separated when the ionic strength of the trans-compartment was maintained at 1 M KCl [31]. We carried out an experiment of different concentrations corresponding to the change of the ionic current to observe the effect of the concentration on the ionic current, which was placed on Additional file 1 (see Figure S1).) Electrodes (Ag/AgCl) were placed in both chambers and connected to the headstage of a patch clamp amplifier (Axopatch 700B, Molecular Devices, Inc., Sunnyvale, CA, USA) which allowed the ionic current to be measured under constant voltage. Signals were acquired at a 50-kHz sampling rate. The amplifier internal low-pass eight-pole Bessel filter was set at 10 kHz. The entire apparatus was placed in a double Faraday cage enclosure on an anti-vibration table.

### Hybridization experiment

The hybridization experiment was realized using a hybridization solution (HB) which contains 50% formamide, 5× SSC (0.75 M sodium chloride, 75 mM sodium citrate), 0.5% SDS, 5 mM potassium phosphate, and 2 μL antifoam A (Sigma-Aldrich, St. Louis, MO, USA) at pH 7.2. It has a measured conductivity of approximately 6 S.m^−1^. The aqueous solutions of perfectly complementary (PC) (0.2 nM) and non-complementary (NC) (0.2 nM) sequences were prepared in carbonic acid buffer solution. A nanopore containing a single-stranded DNA-modified channel was mounted on the conductivity cell. To achieve DNA hybridization, the DNA-modified channel was exposed to NC and PC solutions for 5 h at 30°C. DNA molecules used in our experiments for functionalization and translocation are listed in Table 1.

**Table 1:** **List of DNA molecules used in our experiments for functionalization and translocation**

**Type**	**Sequence**
Probe	5′-TTTTTTTTTTTTGTCATTATGTGCTGCCATATCTACTTCAGA-3′ sequence
Perfectly complementary (pc-DNA)	CGTAGGTACTCCTTAAAGTTAGTATTTTTATATGTAGTT*TCTGAAGTAGATATGGCAGCACATAATGAC*ATATTTGTACTGCGTGTAGTATCAACAACAGTAACAAA
Non-complementary (nc-DNA)	ACATGTCTTATATATTCTTTAAAATTTTCATTTTTATATGTACTGTCACTAGTTACTTGTGTGCATAAAGTCATATTAGTACTGCGAGTGGTATCTACCACAGTAACAAA

## Results and discussion

### Optimization of silanization times

**Figure 3:**
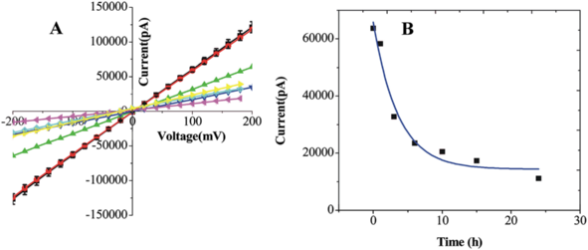
*I*-*V* characteristics and open pore current. **(A)** *I*-*V* characteristics of functionalized nanopore with silanization. Current was measured in 1 M KCl solution (time 0, 1, 3, 6, 10, 15, and 24), respectively. **(B)** Time vs current.

**Figure 4:**
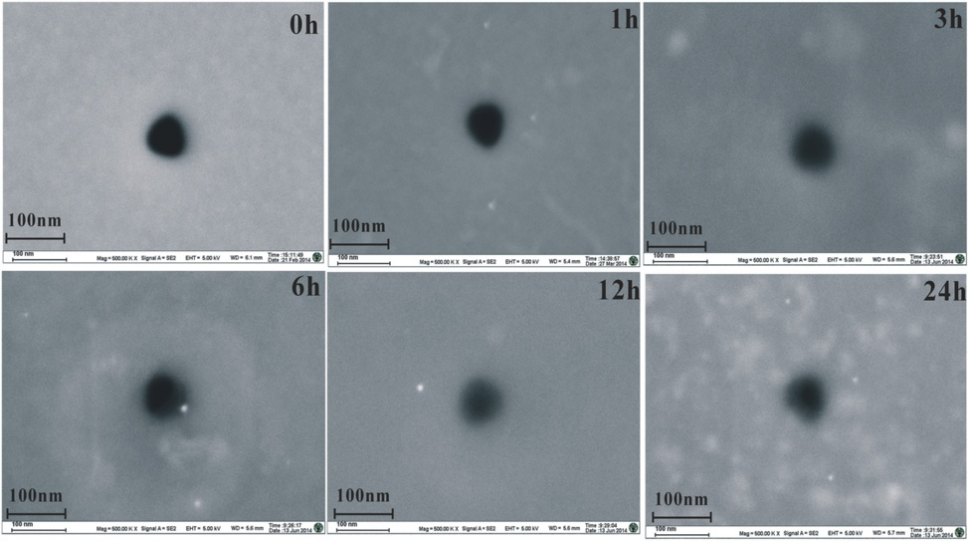
FESEM images. The images were obtained on, respectively, a non-functionalized nanopore (silanization time *t* = 0 h) and five nanopores treated with silanes for 1, 3, 6, 12 and 24 h. The presence of the organic layer is associated with the appearance of a ragged edge around the circular pore and shows time dependence.

The preparation procedure required precise control of the first activation step which was highly dependent on the chosen probe molecules and on the final diameter needed for the specific application, but was extremely simple and effective, enabling a very fast realization of a selective solid-state nanopore biosensor. Therefore, the control of the first step activation time is considerably important. Different nanopores with similar initial diameters (about 60 nm) were functionalized by only varying the duration of the chemical modified. The time (*t*) of exposure to the silane molecules was set between 0 and 24 h. The resizing result of silanization treatment was nicely confirmed by electrical measurements performed on a series of nanopore chips functionalized by using different silanization times. *I*-*V* characteristics of the functionalized nanopore with different silanization times are revealed in the Figure 3A, and an exponential decay was used to estimate the electrical resistance (Figure 3B). As shown in Figure 3B, with the increase of the silanization time, the open pore current gradually reduces. Meanwhile, the values of the open pore current are reported in Figure 3B as a function of the silanization time, allowing us to estimate the continuously growing percentage reduction of the pore area with the thickness of the organic coating. The presence of the organic layer was associated with the appearance of a ragged edge on the circular pore. As evidently shown in Figure 4, it depended on the silanization time. Treatment of 1 h was not sufficient to produce noticeable modifications. However, the silane film started to uniformly cover the nanopore surface at 3 h. For 6, 12, and 24 h, shrinking of the pore diameter was very easy, causing the clogging of the nanopores. Besides, the open pore current will further reduce with successful immobilization of 1,4-phenylene diisothiocyanate and the probe (oligonucleotides, 42 bases about 14 nm) on the inner channel wall. The revealed behavior can be fit by an exponential decay with a single time constant of 3 h, whose value was likely related to temperature conditions used during silanization. This result was consistent with the functionalization which was not initiated with the formation of a simple monolayer of silane molecules [6].

### Characterization of chemical modification of nanopores

The covalent coupling of an amine-terminated DNA probe with the hydroxyl groups (Figure 2B) was achieved by using the following procedure. The entire membrane was activated with 3-APTES that was linked to the hydroxyl groups of the native silicon oxide layer present on the silicon nitride surface. The chip was then treated with the 1,4-phenylene diisothiocyanate cross-linker. Finally, the cross-linker was covalently coupled with the terminal amine on the DNA probe.

**Figure 5:**
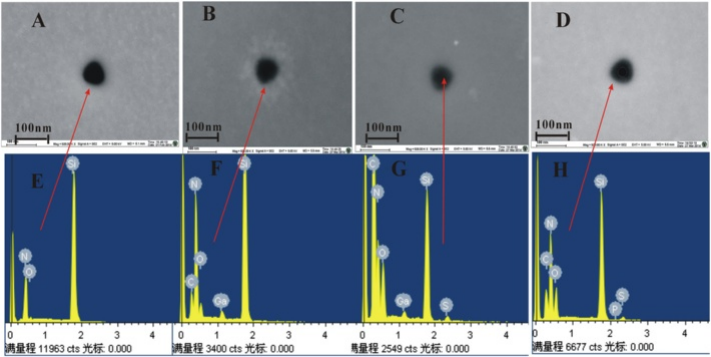
Characterization of functionalized nanopores. **(A-D)** FESEM images: before the functionalization, functionalized with 3-APTES, treated with cross-linker, and DNA modified, respectively. **(E-H)** EDS measurements: before the functionalization (-OH), functionalized with 3-APTES (NH_3_^+^), treated with cross-linker (S = N = C), and DNA modified, respectively.

The immobilization of the probe is another key step for the fabrication of this sequence-specific recognition biosensor. Here, FESEM imaging and EDS were introduced to verify successful immobilization after each step, and the results are shown in Figure 5. Compared with the image of the pore region before the functionalization (Figure 5A), the functionalization layer appeared as a grey shadow in the pore region as shown in Figure 5B,C. Meanwhile, Figure 5E (before the functionalization), Figure 5F (functionalized with 3-APTES), Figure 5G (treated with the cross-linker), and Figure 5H (DNA modified) show the EDS measurements. The original SEM images of Figure 5A,D were placed on Additional file 1 (see Figures S1 and S2). The atomic percentage (at.%) before and after the functionalization of nanopores is listed in Table 2, and after the functionalization, there was 21.81% atomic increase in nitrogen around the pore (1#) compared to that before the functionalization (2#). Besides, a new element C is observed after functionalization of the nanopore, and O (at.%) is also increased from 0.98 to 3.40. The Ga contamination shown in Table 2 (2#, 3#) was due to the ion beam used for both drilling and deposition [32]. The result indicated the existence of 3-APTES in the pore. In addition, we observe new elements S and P from 3# and 4#, respectively, which are determined chemical elements and structures of the cross-linker and DNA. The result confirmed the success of the DNA immobilization on the nanopore.

**Table 2:** **The atomic percentage (at.%) before and after functionalization**

**Number**	**C (at.%)**	**N (at.%)**	**O (at.%)**	**Si (at.%)**	**S (at.%)**	**P (at.%)**	**Ga (at.%)**
1#	-	42.50	0.98	56.53	-	-	-
2#	9.89	51.77	3.40	34.39	-	-	0.54
3#	50.24	24.79	8.39	15.17	1.17	-	0.24
4#	13.63	46.27	9.16	29.58	1.28	0.08	-

1#, before the functionalization; 2#, functionalized with 3-APTES; 3#, treated with cross-linker, 4#, DNA modified; at.%, atomic percent.

**Figure 6:**
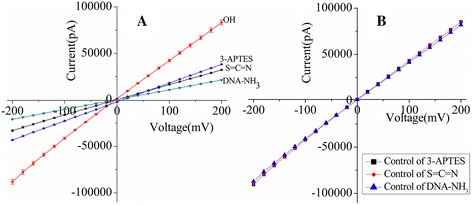
The *I*-*V* curves of nanopores and the control experiment. **(A)** *I*-*V* characteristics of functionalized nanopore with diameters of approximately 60 nm, functionalized with 3-APTES (3 h) and treated with cross-linker (5 h) (S = N = C) and DNA-NH_3_ (overnight 37°C). *I*-*V* curves were measured in 1 M KCl solution. **(B)** The corresponding control experiment.

In order to further characterize layer-by-layer self-assembly on the inner pore wall, *I*-*V* curves of the functionalized nanopore, which originated from their charged surfaces, were measured. Figure 6A shows the *I*-*V* curves of the nanopores used for immobilizing DNA probes on the channel surface. The control experiments were performed to demonstrate that a decrease in current actually stems from the specific surface chemistry in Figure 6B. The ionic currents were recorded under symmetrical electrolyte conditions using 1 M KCl solution. Before modification, the ionic current was measured at a potential difference of 200 mV resulting in an ionic current of 83.2 ± 0.5 nA. After 3-APTES links to the hydroxyl groups, a significant change in the ionic current was observed from Figure 6A (blue). The control experiment of the 3-APTES-modified nanopore was performed. We observed that the ionic current in Figure 6(B) (black) remained unchanged compared to that in Figure 6A (red), which demonstrated that a decrease in current stems from modification of 3-APTES. Then, when the chip was treated with cross-linkers, we discovered a further decrease in the ionic current in Figure 6A (black). Figure 6B (red) showed control of the cross-linker (1,4-phenylene diisothiocyanate), which manifested that a decrease in current stems from modification of the cross-linker. Upon immobilizing DNA probes, a significant decrease in the ionic current was observed under the same applied bias voltage in Figure 6A (green). Meanwhile, the control experiment of immobilizing DNA probes was carried out. We did not observe any significant change in the *I*-*V* characteristics from Figure 6B (blue), which showed that a decrease in current stems from immobilization of DNA probes. In particular, we used 3-APTES, which is highly reactive and could give rise to a number of possible surface structures. The interaction with surface silanol groups (Si-OH) results in siloxane bonding (Si-O-Si), accompanied by ROH as a by-product R = CH_2_CH_3_. It was possible to produce ordered self-assembled monolayers (SAMs) with this surface. However, self-polymerization of 3-APTES in solution usually prevents the formation of homogeneous films, causing polysiloxane deposition at the surface, due to 3D vertical polymerization [6]. This partial vertical polymerization of silane molecules could be interestingly exploited to control the resizing of nanostructures by chemical functionalization. The changes in the *I*-*V* characteristics before and after modification confirmed the successful immobilization of the DNA probe on the inner channel wall.

Considering geometric effects, the change of conductance can be used to determine the approximate thickness of the 3-APTES layer deposited inside the nanopore. With an initial pore diameter of approximately 60 nm, we assume that the diameter of the nanochannel is constant with a fixed length *L* equal to the silicon nitride membrane thickness, and the ‘diameter of modified nanopore’ can be introduced, which is given by Equation 1 [27]:

1 $$ {d}_{MN}=2\sqrt{\frac{L}{\sigma \pi R}} $$

The *σ* (10 S.m^−1^) is the conductivity of 1 M KCl solution at 25°C. The value of the pore resistance (*R*) was obtained by the current measurements. The ‘diameter of modified nanopore’ could be calculated as approximately 34 nm by Equation 1. However, electron microscopy (SEM) characterization showed that the diameter of the modified nanopore is approximately 37 nm, which is mainly attributed to the etching process and chemical modification leading to different shapes of the nanopore. This cylindrical approximation, although useful to analyze the nanopore general properties, can only be used as a reference.

### Nanopore DNA translocation experiments

DNA-modified solid-state nanopores can serve as sensors to detect a single-stranded DNA oligomer with sequence complementary to the immobilized DNA probe.

**Figure 7:**
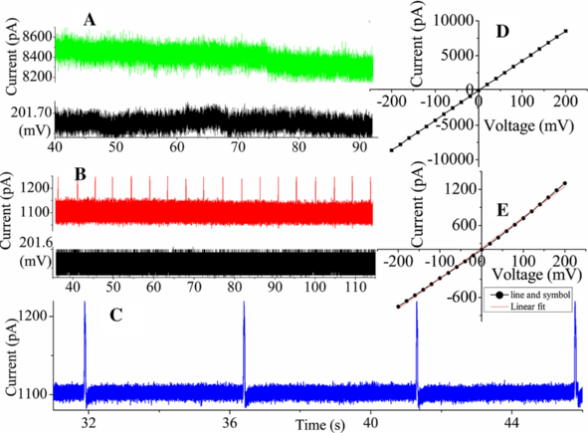
Current trace and *I*-*V* plots. Current trace showing **(A)** non-complementary (green), at 200 mV (black), and **(B)** perfectly complementary (red), at 200 mV (black), hybridization experiment translocations, and **(C)** enlargement of perfectly complementary hybridization experiment translocations in HB solution upon applying a voltage of 200 mV(low-pass eight-pole Bessel filter was set at 5 kHz). *I*-*V* plots after exposing the DNA-modified nanochannel to **(D)** nc-DNA and **(E)** pc-DNA strands in HB solution.

To perform the hybridization process, we performed hybridization experiments on a chemically modified chip using target molecules NC and PC to the probe ones. Figure 7D,E shows the variations in the *I*-*V* plots after exposing the DNA-modified nanochannel to nc-DNA and pc-DNA strands, respectively. Subsequently, the physically adsorbed DNA molecules were removed by thoroughly washing the channel with 10 mM pH 7.4 PBS solution, and *I*-*V* curves were measured in HB solution. We did not observe any significant change in the *I*-*V* characteristics upon exposing the DNA-modified channel to the nc-DNA oligomer. This confirmed the lack of hybridization of nc-DNA, leaving the original DNA surface undisturbed. Subsequently, when the same channel was exposed to the pc-DNA strand, it resulted in a further decrease in the ionic current. From the variations in the *I*-*V* plots, we can conclude that the sensor exhibits a remarkable selectivity and specificity toward the fully complementary sequence compared to the non-complementary.

To demonstrate this option, the control experiment was performed by adding, on the cis side, an HB solution containing, as target molecules, oligonucleotides with the same sequence of those used for the functionalization procedure (0.2 nM). This NC target was left 5 h in the cell before starting the electrical measurements, in order to favor the diffusion of the molecules. Positive voltage (200 mV) was applied, and translocation events were sampled at 100 kHz. We observed no translocation events in Figure 7A, and even if the current signal appeared to be more noisy, no translocation events were detected. This phenomenon may be due to the passage of such short target molecules which is too fast to be detected as a current modulation and the quite large dimension of the functionalized nanopore [23].

The stable functionalization with oligonucleotides also imparts selectivity to the detection mechanism and allows identification of complementary target sequences. To demonstrate this capability, the experiment was then repeated with PC target molecules that were left 5 h in the cell before starting the electrical measurements. The used target concentration is sufficiently high to guarantee that, despite the hybridization with the probes attached to the entire Si_3_N_4_ membrane, enough unhybridized strands remain in the cis chamber and could be driven toward the nanopore when a voltage bias was applied. The result appeared remarkably different in this case. We observed sparse sub-millisecond current blockades caused by molecules translocating through the nanopore in Figure 7B when a voltage bias (200 mV) was applied. At the same time, we also found spike-like current increases. Because the hybridization experiment was realized using HB solution, which has a measured conductivity of approximately 6 S.m^−1^, the conductivity of 0.5 M KCl is about 6 S.m^−1^. It has been reported that DNA translocation is shown to result in either a decrease ([KCl] > 0.4 M) or increase of the ionic current ([KCl] < 0.4 M) [30]. Therefore, we deem that this phenomenon mainly depends on the salinity of the solution. Many different current levels can be distinguished, as evident in the enlargement of Figure 7C (the low-pass eight-pole Bessel filter was set at 5 kHz). The original data of positive voltage (150, 200, and 400 mV) were added in Additional file 1.

**Figure 8:**
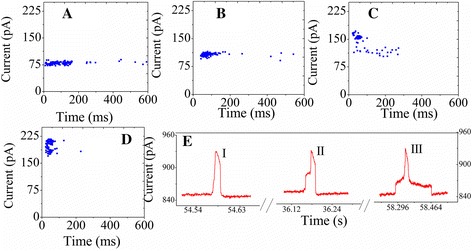
Two-dimensional scatter plots of current versus event duration of DNA PC hybridization experiments. At 1 M KCl HB solution at different voltages **(A)** 150 mV, **(B)** 200 mV, **(C)** 300 mV, and **(D)** 400 mV. **(E)** Three typical events.

Figure 8 shows two-dimensional scatter plots of current versus event duration of DNA PC hybridization experiments at different voltages ((A) 150 mV, (B) 200 mV, (C) 300 mV, and (D) 400 mV). All of the proteins show a cluster of events at the event duration limit imposed by the filter frequency. Outliers with long event durations are observable in most of the scatter plots. We hypothesize that these events are caused by transient adherence of hybridized pc-DNA strands as they translocate, whereas the main event clusters are due to hybridized pc-DNA traveling ballistically through the nanopore.

The current blockage signals revealed the information of the size, conformation, and interactions of DNA passing through the nanopore. We observed three typical current traces for PC target molecules (Figure 8E). Event (I) is unhybridized strands in the cis chamber and could be driven toward the nanopore when a voltage bias was applied. Event (II) shows after a longer duration sticking passing through the nanopore, which is hybridized strands passing through the nanopore. Event (III) is milliseconds in duration and has two levels, suggesting transient adherence of two hybridized molecules to the sides of the nanopore walls.

Here, we would like to mention the recent work reported by Gyurcsanyi and coworkers in which PNA-functionalized gold nanotubes embedded in polymer membranes were applied for the label-free detection of cDNA [23,28,33]. Compared to their work which is also aiming at specific DNA detection, our technique is more straightforward, simpler, and low-cost. Such kinds of functionalized pores have been successfully used to perform long dsDNA translocation experiments, thanks to the sensitivity acquired through the pore size reduction, hybridization with complementary target sequences, and the selectivity of the interaction between the probe attached to the sensor surface and the dispersed target molecules [6].

## Conclusions

In this study, we successfully fabricated a Si_3_N_4_ nanopore using FIB. A simple functionalization procedure was demonstrated to produce an effective single-molecule biosensing device. Our approach was based on the simultaneous pore size tuning and functionalization as induced by the chemical covalent attachment of probe biomolecules on its surface. FESEM imaging, EDS, and electrical measurements were used to show that by varying the silanization time, it is possible to control and tune the functionalization efficiency and shrink the nanopore to the chosen size, while introducing specific sensing probes. We found that by using 42-mer oligonucleotides as probe molecules and 3 h of silanization in ambient temperature, it is possible to obtain approximately 50% size reduction of the pores. The presented data demonstrate the ability of the DNA to selectively detect complementary target sequences, which is to distinguish between molecules depending on their affinity to the functionalization. The DNA-modified channel provided a novel sensing platform which can discriminate between perfectly complementary and non-complementary DNA strands. The biosensor displayed a good selectivity and specificity to a DNA strand with a sequence complementary to the attached DNA probe. The great advantage of the proposed strategy is its simplicity and versatility. Indeed, being adaptable to different probe molecules, it allows the preparation of biosensors for a variety of different applications and target biomolecules. In this context, we believe that on the basis of these findings, one would be able to construct a setup for the detection and identification of an unknown DNA oligomer by using specific DNA sequences in the future.

## Additional file

Additional file 1:
**Supplementary information.** A file showing five supplementary figures.

## Abbreviations

3-APTES: 3-aminopropyltriethoxysilane
BSA: bovine serum albumin
EDS: energy-dispersive X-ray spectroscopy
FESEM: field-emission scanning electron microscopy
FIB: focused ion beam
NC: non-complementary
PC: perfectly complementary
TMAH: tetramethylammonium hydroxide
