# Supplementary material for: DNA-functionalized silicon nitride nanopores for sequence-specific recognition of DNA biosensor
Source: Nanoscale Res Lett. 2015 May 1;10:205. doi: 10.1186/s11671-015-0909-0 (PMC4420758; doi:10.1186/s11671-015-0909-0)
Supplement: Additional file 1: — Supplementary information. A file showing five supplementary figures. [file 11671_2015_909_MOESM1_ESM.docx]

**Additional file 1**

**DNA** **Functionalized Silicon Nitride Nanopores for** **Sequence-Specific Recognition of DNA Biosensor**

Shengwei Tan^a^, Lei Wang^a^, Jingjing Yu^a^, Chuanrong Hou^a^, Rui Jiang^b^, Yanping Li^c^ Quanjun Liu^a^*

a State Key Laboratory of Bioelectronics, School of Biological Science and Medical Engineering, Southeast University, Nanjing 210096, PR China

b Department of Automation, Tsinghua University, Beijing 1000084, PR China

c Jiangxi-OAI Joint Research Institute, Nanchang University, Nanchang 330047, PR China

We carried out experiment of different concentrations corresponding to the change of ion current to observe effect of the concentration for the ionic current. Ion current (I) related to the conductance of the potassium chloride solution$\sigma$ and nanopores diameter ($d_{MN})$ from equation (a) and (b).

$I=\frac{U}{R}$ (a)

$R=\frac{4L}{\sigma\pi d_{MN}}$ (b)

σ (10 S m^−1^) is the conductivity of 1 M KCl solution at 25 ºC. The value of the pore resistance (R) was obtained by the current measurements. $d_{MN}$is Diameter of modified nanopore. with a fixed length L equal to the silicon nitride membrane thickness. Ion current (I) related to the conductance of the potassium chloride solution$\sigma$ and nanopores diameter ($d_{MN})$ from equation (a) and (b).

1M KCl ~10 S.m^-1^

0.5M KCl ~6 S.m^-1^

0.1M KCl ~1 S.m^-1^

Therefore, with the decrease of the concentration, opening current decreased. with the decrease of nanopores diameter, opening current decreased.





Figure S1





Figure S2 Before functionalization





Figure S3 After DNA immobilization

150mV


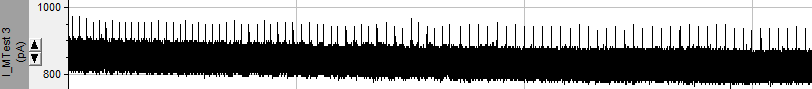


200 mV


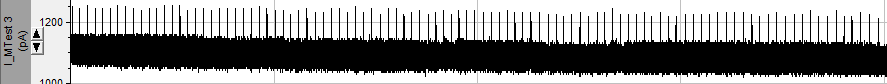


400 mV


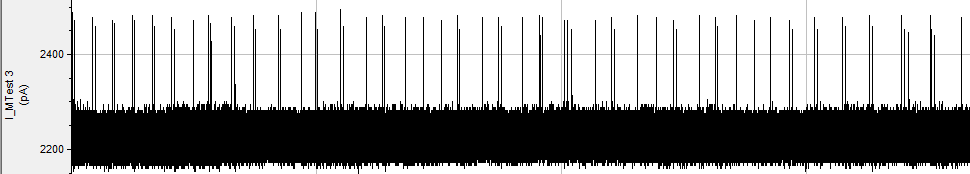


Figure S4

I-V curve collected before nc-DNA insertion was reported in Figure S5 (black). Afterwards, the cell washed several times with water and HB solution. I-V curve collected after washing was reported in Figure S5 (red, before pc-DNA insertion), which demonstrates no residual contamination of the nanopore due to the previous passage of the nc molecules. I-V curves were measured in 1 M KCl solution.





Figure S5
